# The microbiome types of colorectal tissue are potentially associated with the prognosis of patients with colorectal cancer

**DOI:** 10.3389/fmicb.2023.1100873

**Published:** 2023-03-21

**Authors:** Yixin Xu, Jing Zhao, Yu Ma, Jia Liu, Yingying Cui, Yuqing Yuan, Chenxi Xiang, Dongshen Ma, Hui Liu

**Affiliations:** ^1^Department of General Surgery, The Affiliated Hospital of Xuzhou Medical University, Xuzhou, Jiangsu, China; ^2^Department of Pathology, The Affiliated Hospital of Xuzhou Medical University, Xuzhou, Jiangsu, China; ^3^Department of Pathology, Xuzhou Medical University, Xuzhou, Jiangsu, China

**Keywords:** colorectal cancer, tissue microbe, prognostic biomarkers, survival, pathogenic bacteria

## Abstract

As the second leading cause of cancer worldwide, colorectal cancer (CRC) is associated with a poor prognosis. Although recent studies have explored prognostic markers in patients with CRC, whether tissue microbes carry prognostic information remains unknown. Here, by assessing the colorectal tissue microbes of 533 CRC patients, we found that Proteobacteria (43.5%), Firmicutes (25.3%), and Actinobacteria (23.0%) dominated the colorectal tissue microbiota, which was different from the gut microbiota. Moreover, two clear clusters were obtained by clustering based on the tissue microbes across all samples. By comparison, the relative abundances of Proteobacteria and Bacteroidetes in cluster 1 were significantly higher than those in cluster 2; while compared with cluster 1, Firmicutes and Actinobacteria were more abundant in cluster 2. In addition, the Firmicutes/Bacteroidetes ratios in cluster 1 were significantly lower than those in cluster 2. Further, compared with cluster 2, patients in cluster 1 had relatively poor survival (Log-rank test, *p* = 0.0067). By correlating tissue microbes with patient survival, we found that the relative abundance of dominant phyla, including Proteobacteria, Firmicutes, and Bacteroidetes, was significantly associated with survival in CRC patients. Besides, the co-occurrence network of tissue microbes at the phylum level of cluster 2 was more complicated than that of cluster 1. Lastly, we detected some pathogenic bacteria enriched in cluster 1 that promote the development of CRC, thus leading to poor survival. In contrast, cluster 2 showed significant increases in the abundance of some probiotics and genera that resist cancer development. Altogether, this study provides the first evidence that the tissue microbiome of CRC patients carries prognostic information and can help design approaches for clinically evaluating the survival of CRC patients.

## 1. Introduction

The incidence and mortality of colorectal cancer (CRC) have increased significantly in recent years, ranking the 3rd and 5th among all malignant tumors, respectively (Siegel et al., 2020; Zhao et al., 2020; Lu et al., 2021). Most patients are in the middle and late stages when diagnosed, which seriously threatens the survival and quality of life of patients (Dekker et al., 2019; Cienfuegos-Jimenez et al., 2021; Peng et al., 2022). The 5-year relative survival ranges from more than 90% in stage I patients to slightly more than 10% in stage IV patients (Brenner et al., 2014; Biswas et al., 2021). Due to the frequent recurrence and metastasis, the prognosis of CRC is yet to be improved, especially for those with unknown tissue origin (He et al., 2020a,b; Liu et al., 2021). Accurate prediction of the prognosis of CRC patients is of great significance for targeted treatment and avoidance of overtreatment. However, at present, most studies are focused on identifying biomarkers for early screening of CRC (Ahlquist et al., 2000; Tanaka et al., 2020; Wu et al., 2021), and the exploration of biomarkers for patient prognosis is still limited, except for a few initial tries (Yang et al., 2022; Yuan et al., 2022).

Microbial communities are thought to influence the initiation, progression, metastasis, and response to the treatment of a variety of cancers (Cullin et al., 2021; Qi et al., 2022; Wang et al., 2022). In addition to gut microbes, microbes in other niches may influence host physiology. Many members of the microbial community can induce cell proliferation by activating certain signaling pathways. Microbial communities can act as a source of activating signals for aberrant epithelial cell proliferation, initiating cancer (Fulbright et al., 2017). This includes microbes on the outer surface and mucosal sites, as well as tissue-resident microbes (Heymann et al., 2021). Castellarin et al. (2012) found that *Fusobacterium nucleatum* transcripts were 400 times more abundant in CRC tumor tissues than in normal tissues. In addition, *F. nucleatum* has been associated with liver metastases (Bullman et al., 2017), amplifying its potential impact on cancer. *Bacteroides fragilis* is a commensal bacteria active in the whole colon, among which enterotoxigenic *B. fragilis* (ETBF) is believed to be associated with the induction of colitis and colon tumorigenesis due to its enrichment in stool and mucosal samples of cancer patients (Boleij et al., 2015; Haghi et al., 2019). Besides, healthy gut microbes are typically made up of dominant populations of *Lactobacilli*, *Bacteroides*, and *Bifidobacterium* (Nakatsu et al., 2015). In CRC, *Fusobacterium*, *Porphyromonas*, *Parvimonas*, *Peptostreptococcus*, and *Gemella* showed excessive dominance, indicating the occurrence of bacterial flora imbalance (Nakatsu et al., 2015; Wirbel et al., 2019; Cheng et al., 2022). However, there is no consensus that one or more microbes can be associated with the prognosis of CRC patients, whether it is intestinal flora or intratumoral microbes of tumor tissue. Consequently, there is an urgent need to study the association between microbial communities and the prognosis of patients with malignant tumors.

Enterotype is a new concept proposed by Arumugam et al. (2011) in the study of intestinal microbiota in 2011. Arumugam et al. (2011) found that the gut microbiota can be divided into three groups according to the dominant genera, with *Bacteroides*, *Prevotella*, and *Bifidobacteria* as the dominant types. Different enterotypes have different microbiota structures and functional genes, and people with different enterotypes have different ways of energy metabolism and storage. In recent years, more and more studies have shown that a large number of microbes are enriched in tumor tissues (Hu et al., 2017; Nejman et al., 2020; Wong-Rolle et al., 2021). Therefore, we wonder whether the colorectal tumor tissue microbiota of CRC patients can be classified similarly to the gut microbiota and whether this classification carries prognostic information of CRC patients, such as the propensity for recurrence and metastasis as well as survival time.

To this end, we collected colorectal microbiological samples from 533 CRC patients at The Cancer Genome Atlas (TCGA). By characterizing the microbial diversity of all samples, we found that the Shannon index of 533 samples showed bimodal distribution. Therefore, based on the clustering of tissue microbiota from all CRC patients, we obtained colorectal tissue microbiota typing. Further, we correlated tissue microbiota typing with prognosis in CRC patients and found that increased relative abundance of certain microbes was significantly associated with worse or better prognosis. This study provides new insights into inferences about the prognosis of CRC patients based on the composition of the dominant bacteria in the tissue microbiota.

## 2. Materials and methods

### 2.1. Data collection and preparation

A total of 533 tissue microbiome samples of CRC patients and the corresponding metadata were obtained in this study. Cancer microbiome data and the clinical metadata data used in this study were available at ftp://ftp.microbio.me/pub/cancer_microbiome_analysis/ (Poore et al., 2020). The microbial abundance matrix in the data set was annotated by two methods, Kraken and Shotgun. Given Kraken’s high usage rate in metagenomic analysis, only the microbial abundance obtained from Kraken’s annotation was used in this study. Microbiome data included six levels of microbial count including kingdom, phylum, class, order, family, and genus. We calculated the relative abundance of microbes at each level for subsequent analysis.

### 2.2. Clustering analysis

Based on the tissue microbiome abundance matrix, all samples were clustered using the “partitioning around medoids” (PAM) clustering method. Clustering was conducted with package “cluster” in R. Different from K-means clustering based on means, PAM is based on more robust partitioning around central points. In this study, we obtained five groups based on the microbial community at the phylum level by PAM clustering. To reduce the complexity and improve the rationality of the analysis, we further combined these five groups into two groups with significant differences in tissue microbes.

### 2.3. Survival analysis and dimension reduction

The overall survival between different groups was compared by Kaplan–Meier (KM) analysis, and the *p* value was generated with the log-rank test. In this study, we divided all samples equally into two groups (High vs. Low) based on the relative abundances of Proteobacteria, Actinobacteria, Firmicutes, and Bacteroidetes, respectively. Then, survival analysis was conducted on these two groups. Principal component analysis (PCA) was performed with packages “FactoMineR” and “factoextra” in R. The *R*^2^ and *p* value were calculated by an ANOSIM test. Univariate cox regression was performed by the R package “survminer.”

### 2.4. Linear discriminant analysis effect size analysis

Linear discriminant analysis effect size (LEfSe) (Segata et al., 2011), an analytical tool for discovering and interpreting high-dimensional data biometrics (genes, pathways, taxons, etc.) was used to determine the significantly different genera in relative abundance between the two clusters. LEfSe used linear discriminant analysis (LDA) to estimate the magnitude of the effect of the abundance of each component (species) on the differential effect. In this study, we identified 11 potential biomarkers at the genus level with an LDA score > 4 and *p* < 0.05.

### 2.5. Network analysis

We mapped the co-occurrence network of tissue microbiota in two groups of colorectal cancer patients. Correlation coefficients and *p* value between the microbes at the phylum level were generated by the R function “rcorr” in the “Hmisc” package. Further, the network was visualized by Gephi (Bastian et al., 2009), a software tool for building and visualizing bibliometric networks. Only the correlation *p*-values less than 0.01 were shown in the network. The network graph showed only edges with correlation coefficients greater than 0.2 and less than −0.2. Nodes in the network diagram represent microbes, and edges represent correlations between microbes. Node size indicates the relative abundance of microbes. The microbes whose names are shown in the network diagram are the important ones in the network, namely the nodes with a high degree.

## 3. Results and discussion

### 3.1. Tissue microbe profiles of colorectal cancer patients

**Figure 1:**
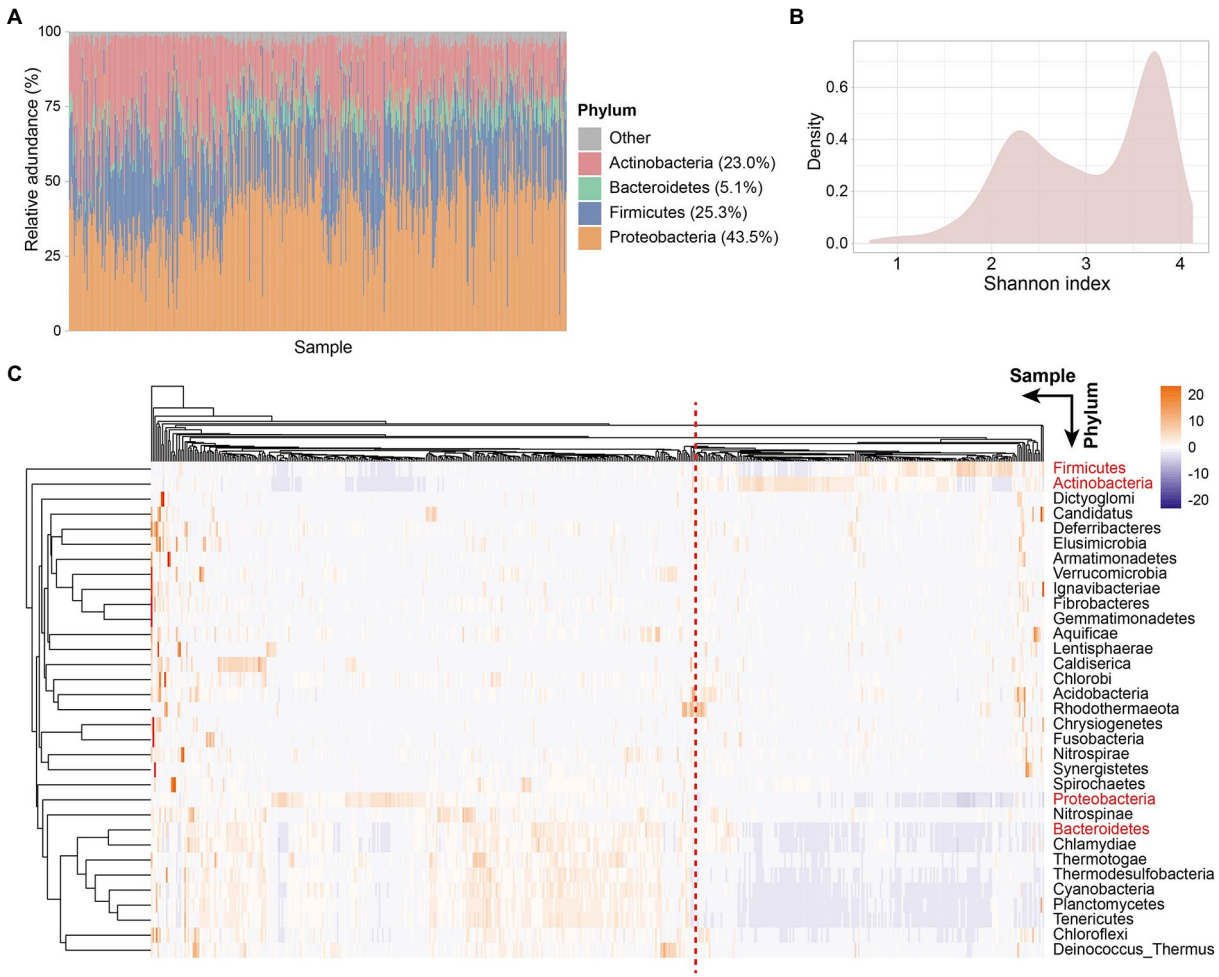
Tissue microbe profiles of CRC patients. **(A)** Tissue microbial community composition at phylum level across all samples. Different color represents different phyla. The four phyla with the highest relative abundance are shown in the figure. Each column represents a sample. **(B)** Density plot of Shannon index of all samples. **(C)** Clustering heatmap based on the relative abundance of 33 species at the phylum level in all samples. Rows represent species and columns represent samples. The names of the four phyla with the highest relative abundance are shown in red. Samples separated by red dashed lines differed in relative abundance at the phylum level species.

Colorectal tissue has a different microbiota profile than the gut. Proteobacteria was the phylum with the highest relative abundance in CRC patient tissues with an average relative abundance of 43.5%, followed by Firmicutes, Actinobacteria, and Bacteroidetes, with the relative abundance of 25.3, 23.0, and 5.1%, respectively (Figure 1A). Similarly, the dominant flora in the gut is mainly composed of Firmicutes, Bacteroidetes, Actinobacteria, and Proteobacteria, accounting for more than 97% of the intestinal flora (Eckburg et al., 2005). However, different from tissue microorganisms, the dominant phyla of gut microbiota are Firmicutes and Bacteroidetes, with only a small proportion of other phyla (Stopinska et al., 2021). An increase in Proteobacteria in the gut is considered a microbial marker of dysregulation of the gut microbiota and a potential diagnostic feature of disease risk (Shin et al., 2015). We detected a high abundance of Proteobacteria in the tissues of CRC patients, which also represents the deterioration of colorectal tumors in patients.

Next, to explore the microbial diversity of CRC patients’ tissues, we calculated the Shannon index of all samples. Notably, the distribution of microbial diversity was bimodal (Figure 1B), with a smaller peak at 2.3 and a larger peak at 3.7. Further, we created a clustering heatmap based on the abundance matrix of phylum-level microbes for all samples (Figure 1C). Similarly, all samples could be clustered into two main groups based on phylum-level microbes across the samples. Preliminarily, we found that the abundances of the four dominant phyla (Proteobacteria, Firmicutes, Actinobacteria, and Bacteroidetes) in the tissues showed differences between the two groups. The large differences in the abundance of dominant phyla led us to wonder whether the tissue microbiota of CRC patients is classified as similar to the enterotype of gut microbiota.

### 3.2. CRC patients can be divided into two clusters based on tissue microbiome, and the prognosis of the two clusters is significantly different

**Figure 2:**
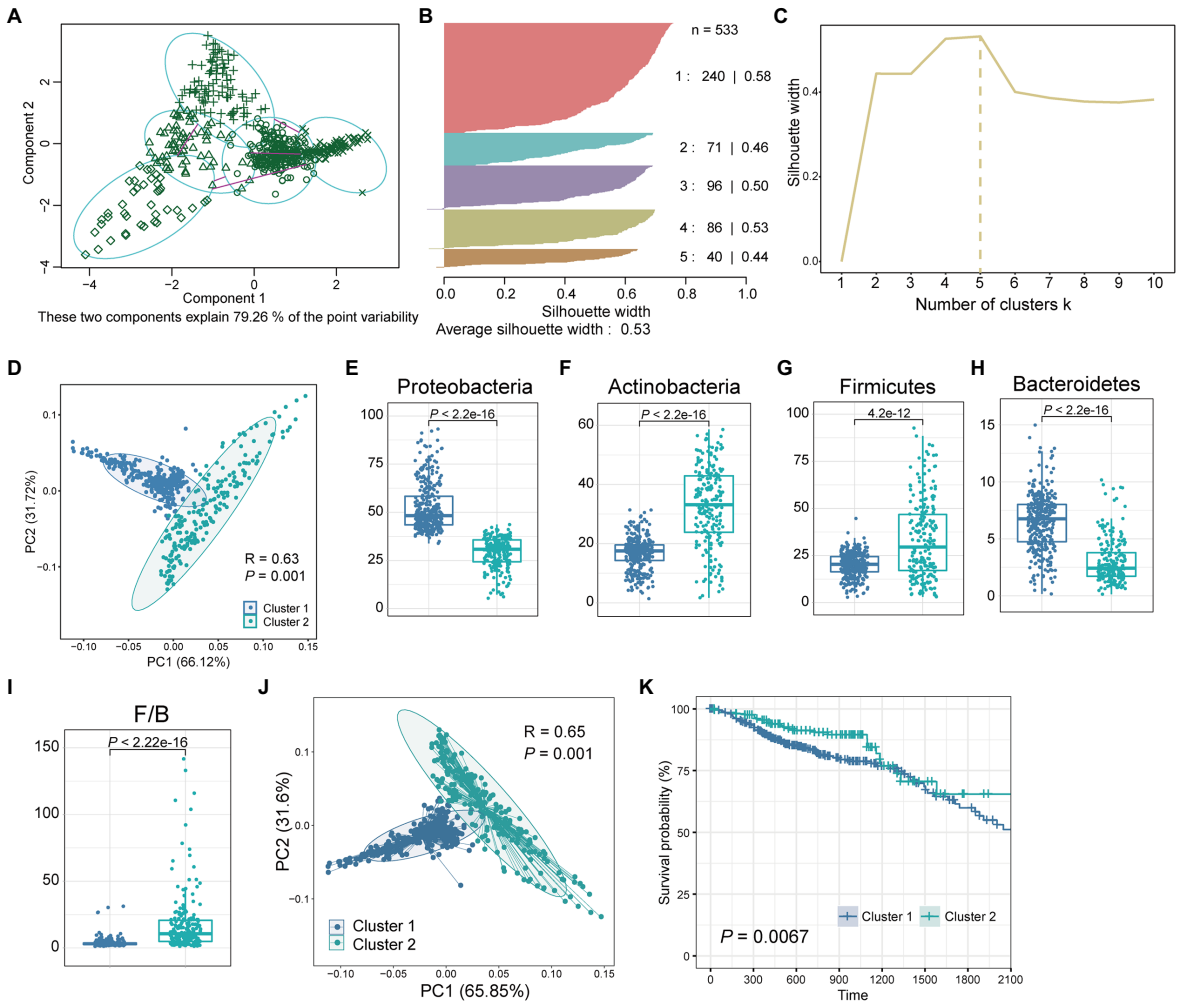
Two clusters obtained by clustering the tissue microbiome abundance. **(A)** All samples were clustered into five groups by PAM clustering method. These two components explain 79.26% of the point variability. **(B)** The silhouette width and sample size of the five groups. **(C)** The corresponding silhouette width when the clustering number is 1–10. **(D)** PCA plot of relative abundance of four dominant phyla for CRC samples reveals considerable variation between cluster 1 and cluster 2. The *R*^2^ and *p* value was calculated by an ANOSIM test. Boxplot of differences in **(E)** Proteobacteria, **(F)** Actinobacteria, **(G)** Firmicutes, **(H)** Bacteroidetes, and **(I)** Firmicutes/Bacteroidetes (F/B) between cluster 1 and cluster 2. The *p* value was calculated by a Wilcoxon rank-sum test. **(J)** PCA plot of relative abundance of tissue microbe data at the phylum level for CRC samples reveals considerable variation between cluster 1 and cluster 2. **(K)** Kaplan–Meier survival curve for overall survival of cluster 1 and cluster 2. The *p* value was calculated by log-rank test.

We next investigated whether the tissue microbiome abundance reflected the same bimodal distribution as observed for the Shannon index. For this, we used a clustering method called “partitioning around medoids” (PAM) for the abundance of the four dominant phyla with the highest relative abundance. The clustering results showed that all samples were divided into five groups with silhouette widths of 0.58, 0.46, 0.50, 0.53, and 0.44, respectively (Figures 2A,B). We further verified the clustering quality with silhouette width, and the result showed that the silhouette width was the highest (0.53) with *k* = 5, suggesting that was the optimal number of clusters (Figure 2C). These two components explain 79.26% of the point variability. Besides, considering the bimodal distribution presented by the Shannon index of all samples (Figure 1B) and the clear two groups presented by clustering heatmap (Figure 1C), we further combined these five groups into two clusters according to the patient survival. Finally, we obtained two clusters of the five groups, with significant differences (*p* = 0.0067) in survival between the two clusters (Supplementary Table S1). PCA showed that the relative abundance of the four dominant phyla of the two clusters was significantly different (Figure 2D; ANOSIM, *p* = 0.001, *R*^2^ = 0.63). Besides, consistent with the bimodal distribution (Figure 1B), the Shannon index of cluster 1 was significantly higher than that of cluster 2 (Supplementary Figure S1). In-depth, we compared the differences of single species between the two clusters separately. Results showed that the relative abundance of Proteobacteria, Actinobacteria, Firmicutes, and Bacteroidetes were significantly differences between cluster 1 and cluster 2 (Figures 2E–H, Wilcoxon test, *p* < 4.2e-12). Specifically, the relative abundance of Proteobacteria and Bacteroidetes in cluster 1 was significantly higher than that in cluster 2, while Actinobacteria and firmicutes were significantly enriched in cluster 2 compared with cluster 1. Besides, the Firmicutes/Bacteroidetes (F/B) ratios of cluster 2 were significantly higher than that of cluster 1 (Figure 2I). The low F/B ratio in the gut is usually considered a biomarker of obesity in humans and animals (Magne et al., 2020). Studies have found reduced F/B ratios in the gut in patients with a variety of diseases, including Alzheimer’s disease, cholelithiasis, and rheumatoid arthritis (Grigor'eva, 2020; Artacho et al., 2021; Sheng et al., 2021). Consequently, we hypothesized that the reduced F/B ratio in colorectal tissues of CRC patients in cluster 1 may affect the tumorigenesis process and thus change the prognosis.

Next, we investigated whether there were differences in prognosis, such as survival, among CRC patients in the two clusters. Since these two clusters were obtained based on the four dominant phyla with the highest relative abundance, we further compared the overall microbial communities of these two clusters. The results showed that the overall tissue microbial communities of cluster 1 and cluster 2 were also significantly different (Figure 2J; ANOSIM, *R*^2^ = 0.65, *p* = 0.001). Then, the survival analysis of patients in these two clusters showed that compared with cluster 2, patients in cluster 1 had significantly worse survival (Figure 2K, *p* = 0.0067). Besides, to verify the computational stability of our results, we randomly selected 50% of the samples and repeated PAM clustering and survival analysis (Supplementary Figure S2). Repeated analysis based on a 50% sample size confirmed the consistency of the results. A significant difference in survival between the two groups could still be found even when the sample size was reduced.

Our results demonstrate that tissue microbiota in CRC patients potentially influences tumor development and that tissue microbiota characteristics carry patient prognostic information.

### 3.3. Microbes with significantly different abundance are responsible for the differentiation of prognosis between the two groups

**Figure 3:**
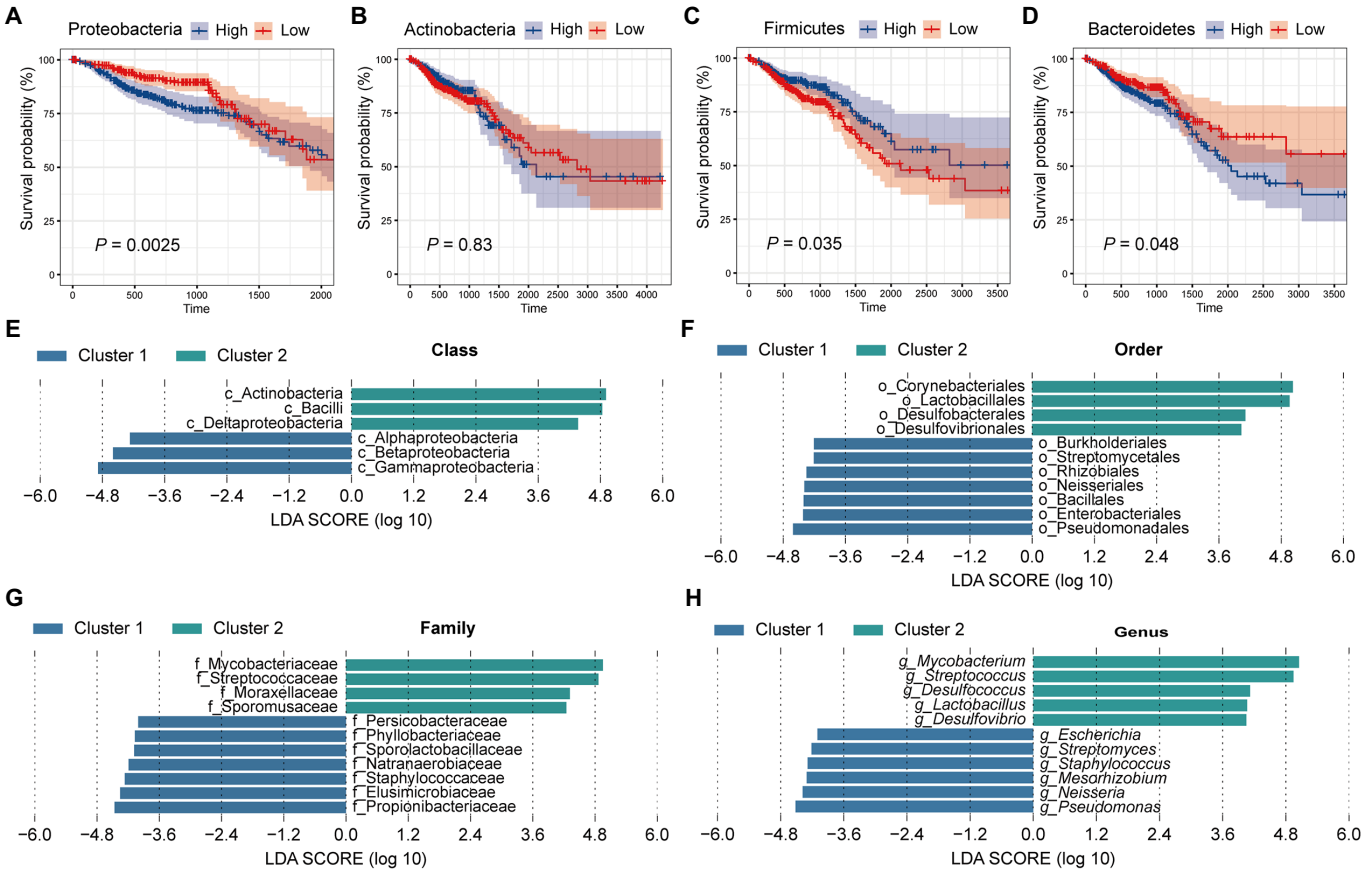
Tissue microbes are responsible for the significant difference in survival between cluster 1 and cluster 2. All samples were divided into two groups based on the relative abundance of **(A)** Proteobacteria, **(B)** Actinobacteria, **(C)** Firmicutes, and **(D)** Bacteroidetes, respectively, and survival curves were performed based on these two groups. The *p* value was calculated by log-rank test. LEfSe identified the significantly different species in relative abundance between the two clusters at the **(E)** class, **(F)** order, **(G)** family, and **(H)** genus level, respectively. The LDA threshold is set to 4.

Previously, we found that colorectal microbiota may affect the prognosis of CRC patients. Next, we focused on which microbiota plays a role in tumor progression. For this, all samples were equally divided into two groups (High and low) according to the relative abundance of the four dominant phyla (Proteobacteria, Actinobacteria, Firmicutes, and Bacteroidetes). Then, we performed survival curves for the two groups, respectively, and compared them (Figures 3A–D). Survival analysis showed that patients with a high abundance of Proteobacteria in colorectal tissue had significantly worse survival (Figure 3A, *p* = 0.0025). In contrast, patients with a high abundance of Firmicutes had significantly improved survival compared with patients with fewer Firmicutes in colorectal tissue (Figure 3C, *p* = 0.035). Similar to Proteobacteria, patients with more abundant Bacteroidetes had significantly better survival (Figure 3D, *p* = 0.048). Among the four dominant phyla, only Actinobacteria do not affect the survival of CRC patients through their actions (Figure 3B, *p* = 0.83). In conclusion, the significantly lower survival rate of patients in cluster 1 compared with patients in cluster 2 is most likely due to the high abundance of Proteobacteria and Bacteroidetes, as well as the low abundance of Firmicutes in tissue microbes of patients in cluster 1.

A study showed that with the development of health-polyp-adenomas-CRC, the relative abundance of Proteobacteria increased gradually, while the relative abundance of Firmicutes decreased gradually (Liu et al., 2020). A comparative analysis of bacterial phyla levels between groups in 40 samples showed a significant increase in Proteobacteria abundance and a significant decrease in Firmicutes in colorectal cancer tissue compared with normal intestinal mucosa (Yang et al., 2019). Liu et al. (2022) found that Proteobacteria had a positive promoting effect on the risk of colorectal cancer and other diseases. Besides, one study confirmed that compared with healthy individuals, inflammatory bowel disease (IBD) and CRC patients had reduced bacterial diversity and abundance, and significantly enriched Bacteroidetes (Quaglio et al., 2022). While our results are consistent with previous studies, more depth, our data suggest that increased Proteobacteria and Bacteroidetes, and decreased Firmicutes in colorectal tissue may be accompanied by poorer patient survival.

### 3.4. Genera belonging to these dominant phyla showed significant differences in abundance between the two clusters of patients

Having found significant differences in tissue microbial composition between the two clusters at the phylum level, we next aimed to explore the similarities and differences between the two groups at other levels. For this, LEfSe analysis with a linear discriminant analysis (LDA) threshold of 4 was used to identify significantly different species in the two clusters (Figures 3E–H). First, at the class level, 6 significantly different species were identified. Specifically, Alphaproteobacteria, Betaproteobacteria, and Gammaproteobacteria were significantly enriched in cluster 2, while Deltaproteobacteria and Bacilli were more abundant in cluster 1 (Figure 3E). At the order level, we identified 7 species enriched in cluster 1 and 4 species enriched in cluster 2. Lactobacillales was more abundant in cluster 2 and Burkholderiales and Enterobacteriales were enriched in cluster 1 (Figure 3F). At the family level, Staphylococcaceae, Propionibacteriaceae, and Elusimicrobiaceae were enriched in cluster 1, while Mycobacteriaceae and Streptococcaceae were enriched in cluster 2 (Figure 3G). At the genus level, a total of 11 significantly different genera were identified, of which 5 were significantly enriched in cluster 2 and 6 were significantly more abundant in cluster 1 (Figure 3H). It has been proposed that *Mycobacteria* as non-specific immune enhancers may have the potential to be effective agents for the prevention or treatment of gastrointestinal diseases, including CRC (Kim et al., 2022). The researchers indicated that heat-killed *Mycobacteria tuberculosis* had a protective effect in a model of inflammation-associated CRC. Meanwhile, we found that *Mycobacteria* were significantly enriched in the tissues of cluster 2 patients, and the better survival of cluster 2 patients confirmed this conclusion. Li et al. (2021) demonstrated that co-culture with *Streptococcus thermophilus* or its conditioned medium reduced the proliferation of CRC cells in culture, and oral gavage of *S. thermophilus* significantly reduced tumorigenesis. *Streptococcus*, a genus belonging to the phylum Firmicutes, similarly showed a significant increase in abundance in cluster 2 patients compared with cluster 1. *Lactobacillus*, a genus belonging to Firmicutes, was found to be significantly more abundant in cluster 2 patient tissues. *Lactobacillus* has long been considered an important probiotic for gut health. Studies have suggested that *Lactobacillus gallinarum* prevented intestinal tumors by producing protective metabolites that promoted CRC cell apoptosis (Sugimura et al., 2021). Besides, the F/B ratio in obese mice was reduced by the treatment of *Lactobacillus sakei* NR28 and *Lactobacillus rhamnosus* GG (Stojanov et al., 2020). In a human clinical trial, the beneficial influence of *Lactobacillus salivarius* was demonstrated (Larsen et al., 2013). Besides, univariate cox regression analysis was performed for the genera with the top 30 relative abundance (Supplementary Figure S3). Among them, four genera (*Escherichia*, *Streptococcus*, *Pseudomonas*, and *Bacteroides*) were significantly correlated with patient survival, which was consistent with KM survival analysis (Figure 3H). What’s more, the four genera belong to Proteobacteria, Firmicutes, and Bacteroidetes, which was also consistent with our PAM clustering.

Most of the genera significantly enriched in the tissues of cluster 1 patients were pathogenic bacteria of CRC or harmful to intestinal health. For instance, recent studies have identified *Escherichia coli*, a species belonging to *Escherichia*, as one of the candidate pathogens for CRC (Cheng et al., 2020). A metabolomic and 16S microbiome analysis of 224 stool samples showed a significant increase in *Staphylococcus* in CRC patients (Clos-Garcia et al., 2020). Besides, the relative abundance of *Enterococcus* and *Neisseria* was significantly higher in the fecal microbiota of patients with invasive cancer compared with early cancer. The genus *Pseudomonas* contains a series of pathogens, among which *Pseudomonas aeruginosa* is a common opportunistic pathogen, which is a common nosocomial infection pathogen in patients with immune deficiency (Mielko et al., 2019). The abnormal proportion of *Pseudomonas nucleomonas* produced a proinflammatory microenvironment, promoted the proliferation of CRC cells, and promotes the chemotherapy resistance of CRC (Chen et al., 2022). *Neisseria meningitides*, an aerobic gram-negative diplococcus, contribute to high morbidity in young adults through an epidemic or sporadic meningitis (Rouphael and Stephens, 2012). Taken together, our data demonstrate that the tissue microbes of CRC patients in cluster 1 tend to enrich some pathogenic bacteria that promote the development of CRC, thus leading to poor survival, while patients in cluster 2 have significantly more bacteria that resist the development of tumors.

### 3.5. The two clusters of patients had different tissue microbiome co-occurrence network properties

**Figure 4:**
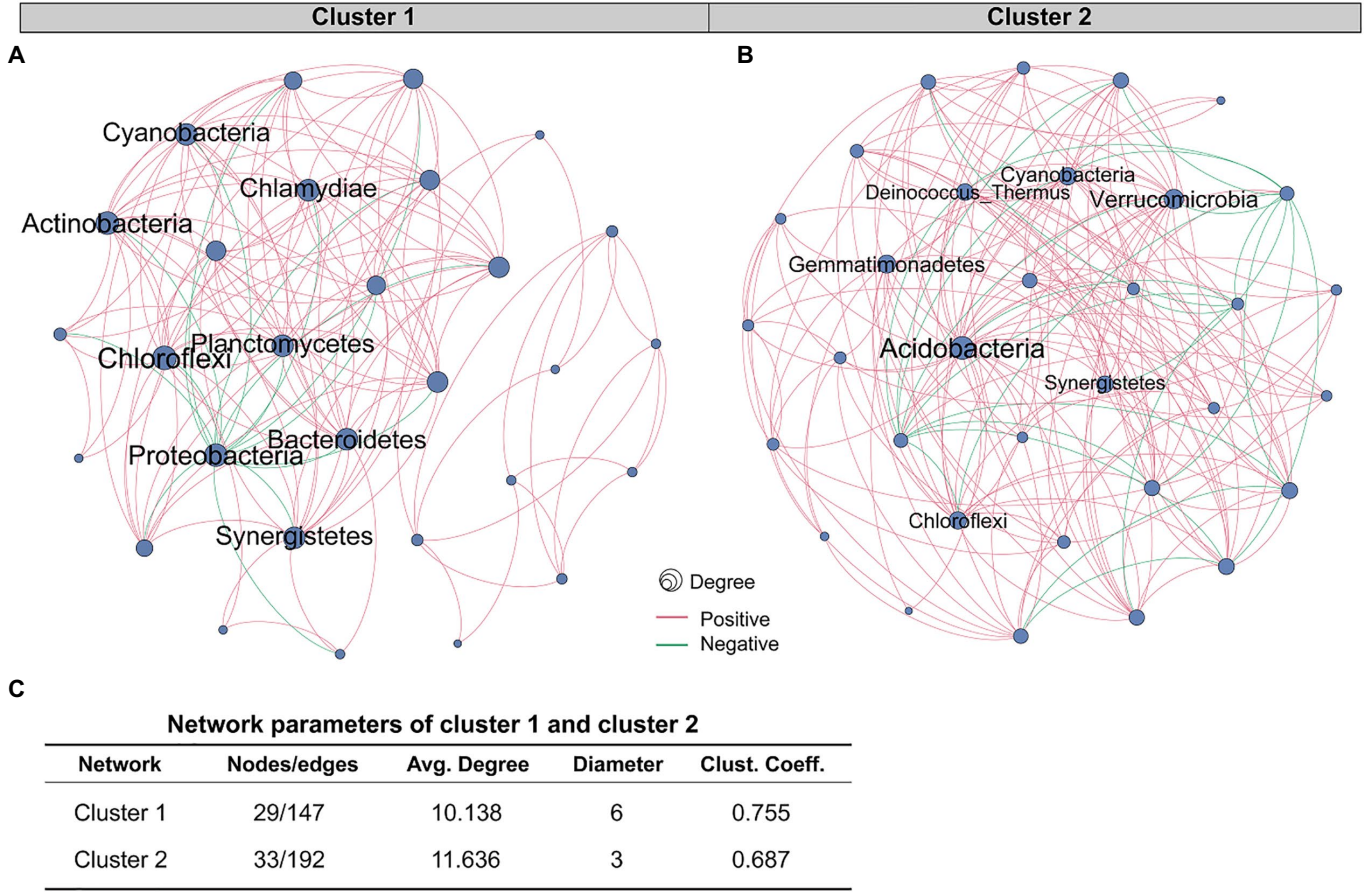
Comparison of co-occurrence network structure and properties between cluster 1 and cluster 2. Co-occurrence networks based on correlation between species at the phylum level of **(A)** cluster 1 and **(B)** cluster 2. The red and green edges represent positive and negative correlations, respectively. Node size is proportional to the relative abundance of species. **(C)** Comparison of co-occurrence network properties between cluster 1 and cluster 2. Avg. Degree, Average degree. Clust. Coeff., Clustering coefficient.

The role of a single or single class of microbes in affecting the occurrence and development of tumors is limited, and the synergistic or antagonistic effects of sufficient species in the microbial community cannot be ignored. Therefore, we constructed co-occurrence networks for the two clusters based on the correlation between species at the phylum level (Figures 4A,B). Network analysis revealed that the nodes and edges of cluster 1 were 29 and 147, respectively, while for cluster 2, they were 33 and 192. For cluster 1, the positive and negative correlations between phylum species were 11.6 and 88.4%, respectively, while for cluster 2, they were 13.0 and 87.0%, respectively. The proportion of positive and negative correlations between tissue microbes in the two clusters was similar. Further, we compared other important network properties between the two clusters, including average degree, diameter, and clustering coefficient (Figure 4C). The results showed that the diameter and clustering coefficient of cluster 1 (6 and 0.755, respectively) were higher than those of cluster 2 (3 and 0.687, respectively), while the average degree of cluster 2 (11.636) was higher than that of cluster 1 (10.138). The important species in the two networks, namely keystone, were significantly different (Table 1). In cluster 1, Chloroflexi, Proteobacteria, and Actinobacteria occupied an important position in the network. However, the keystone species in the network were Acidobacteria, Verrucomicrobia, and Gemmatimonadetes. Besides, compared with cluster 1, the keystone in cluster 2 had a higher degree and weight.

**Table 1:** Comparison of co-occurrence network properties between cluster 1 and cluster 2.

	Phylum	Degree	Eccentricity	Closeness centrality	Betweenness centrality	Clustering
Cluster 1	Chloroflexi	18	5	0.518519	31.274242	0.69281
	Proteobacteria	17	5	0.509091	15.065909	0.772059
	Actinobacteria	17	5	0.509091	17.482576	0.772059
	Bacteroidetes	16	5	0.5	10.774242	0.833333
	Chlamydiae	16	5	0.5	6.274242	0.858333
	Cyanobacteria	16	5	0.5	6.274242	0.858333
	Planctomycetes	16	5	0.5	6.274242	0.858333
	Synergistetes	16	5	0.5	19.848485	0.758333
Cluster 2	Acidobacteria	25	2	0.820513	121.520854	0.326667
	Verrucomicrobia	20	2	0.727273	36.331713	0.542105
	Gemmatimonadetes	18	2	0.695652	45.134963	0.424837
	Chloroflexi	17	2	0.680851	16.273766	0.661765
	Cyanobacteria	17	2	0.680851	20.251597	0.654412
	Deinococcus_Thermus	16	2	0.666667	9.267849	0.741667
	Synergistetes	16	2	0.666667	12.196489	0.691667

The degree represents the number of all edges connected by each node. Closeness centrality represents the sum of the number of nodes that a node can reach divided by the shortest path that can reach the node. Betweenness centrality indicates the ratio between the number of betweenness paths passed by a node by other nodes and the total number of shortest paths in the figure. The eccentricity represents the largest shortest path that a node can reach.

Our study demonstrated that compared with cluster 1, the network of cluster 2 was more complicated. Microbial communities in tumor tissues are not merely collections of independent individuals, but interconnected complexes that communicate, recombine, and coevolve with each other (Layeghifard et al., 2017). Yuan et al. (2022) compared the tissue microbiological co-occurrence networks in 134 lung cancer patients without recurrence or metastasis (non-RM) and 174 patients with recurrence or metastasis (RM) and found that the co-occurrence network of non-RM was more complicated than RM. Recurrence and metastasis as well as survival in our study are both important prognostic indicators of cancer patients (Usuda et al., 2014). Our study shows that the poorer survival of CRC patients is accompanied by a microbiome co-occurrence network of reduced complexity in tissues.

There are several limitations in this study. First, this cohort of 533 CRC patients included confounding factors such as race, country, sex, and age. A recent study looked at the intratumoral microbiota of different cancer types to better understand the influence of age, sex, body mass index (BMI), and ethnicity on the composition of the intratumoral microbiota (Luo et al., 2022). The authors found that race was strongly associated with microbiota abundance, while age, sex, and BMI had little to do with it. Consequently, further analyses should be conducted to distinguish patients of different races and to more accurately identify biologically meaningful microbial markers. A study divided patients with CRC into proximal and distal (Jin et al., 2021), which are not considered the same disease. They found differences in the association of microbes with these two subtypes in CRC patients. For instance, in patients with proximal colon cancers, a high abundance of Fusobacteria was associated with poor prognosis, but not in patients with distal CRC. However, in our study, we did not detect a significant association between Fusobacteria and patient survival. The possible reason is that there are many subtypes of colorectal cancer, and different subtypes may have different associations with tissue microbes. Second, though we show that clusters based on tissue microbiome are associated with survival, we did not provide any prediction model using related microbes. In the future, it will be interesting to develop microbe-based prognosis models. Third, recent studies suggested that tissue histopathological image is correlated with the prognosis of cancers (Liu et al., 2022; Yang et al., 2022; Yao et al., 2022). It would be interesting to study the relationship between tissue microbes and histopathology. Finally, the lack of a healthy control cohort in this study adds a barrier to further understanding changes in tissue microbiota abundance between CRC patients and the normal population. However, tissue from perfectly healthy populations is extremely difficult to obtain, so for colorectal cancer, future studies could consider a control cohort of patients with other intestinal diseases that do not significantly alter the microbial composition of colorectal tissue.

## 4. Conclusion

The present study advances the understanding of the colorectal microbiota in CRC patients, providing evidence for the critical role of tissue microbes influencing the prognosis of patients *via* the variation of the proportion of probiotics, pathogens, or bacteria that can alter the progression of CRC. Moreover, it provides one possible explanation for the heterogeneity of postoperative survival in CRC patients, such that differences in microbial community composition in colorectal tumor tissues of different patients. Thus, we recommend that before the treatment of CRC patients, it is considered to obtain the microbial content of the tumor tissue of the patients to determine the survival time and other prognosis index of the patients, and to assist clinicians in making accurate decisions to avoid overtreatment. Extrapolating from this concept, we suggest that for CRC therapy to be beneficial it needs to be coupled to the tissue microbiome profile of patients.

## Data availability statement

Publicly available datasets were analyzed in this study. This data can be found here: ftp://ftp.microbio.me/pub/cancer_microbiome_analysis/.

## Author contributions

DM and HL contributed to conception and design of the study. YY organized the data. JZ performed the statistical analysis. YX wrote the first draft of the manuscript. YM revised the manuscript. JL, YC, and CX wrote sections of the manuscript. All authors contributed to manuscript revision, read, and approved the submitted version. All authors contributed to the article and approved the submitted version.

## Conflict of interest

The authors declare that the research was conducted in the absence of any commercial or financial relationships that could be construed as a potential conflict of interest.

## Publisher’s note

All claims expressed in this article are solely those of the authors and do not necessarily represent those of their affiliated organizations, or those of the publisher, the editors and the reviewers. Any product that may be evaluated in this article, or claim that may be made by its manufacturer, is not guaranteed or endorsed by the publisher.
