# Supplementary material for: The microbiome types of colorectal tissue are potentially associated with the prognosis of patients with colorectal cancer
Source: Front Microbiol. 2023 Mar 21;14:1100873. doi: 10.3389/fmicb.2023.1100873 (PMC10072283; doi:10.3389/fmicb.2023.1100873)
Supplement: Supplementary file 1 [file Data_Sheet_1.docx]

**The microbiome types of colorectal tissue are potentially associated with the prognosis of patients with colorectal cancer**

Yixin Xu^1^, Jing Zhao^2^, Yuqing Yuan^2^, Jia Liu^2^, Yingying Cui^2^, Chenxi Xiang^2^, Hui Liu^2,3*^, Dongshen Ma^2*^

^1^Department of General Surgery, The Affiliated Hospital of Xuzhou Medical University, Xuzhou, Jiangsu, 221006, China

^2^Department of Pathology, The Affiliated Hospital of Xuzhou Medical University, Xuzhou, Jiangsu, 221006, China

^3^Department of Pathology, Xuzhou Medical University, Xuzhou, Jiangsu, 221004, China

*Correspondence: Hui Liu, Email: hliu@xzhmu.edu.cn

Dongshen Ma, E-mail: madongshen89@163.com

Table S1. Five groups of PAM clustering were randomly grouped into two groups, and the difference in survival between the two groups was tested.

| **Group 1** | **Group 2** | p.value (survival) |
| --- | --- | --- |
| 1 (240) | 2+3+4+5 (293) | 0.3 |
| 1+2 (311) | 3+4+5 (222) | 0.96 |
| 1+3 (336) | 2+4+5 (197) | 0.11 |
| **1+4 (326)** | **2+3+5 (207)** | **0.0067** |
| 1+5 (280) | 2+3+4 (253) | 0.96 |
| 1+2+3 (407) | 4+5 (126) | 0.62 |
| 1+2+4 (397) | 3+5 (136) | 0.43 |
| 1+2+5 (351) | 3+4 (182) | 0.25 |


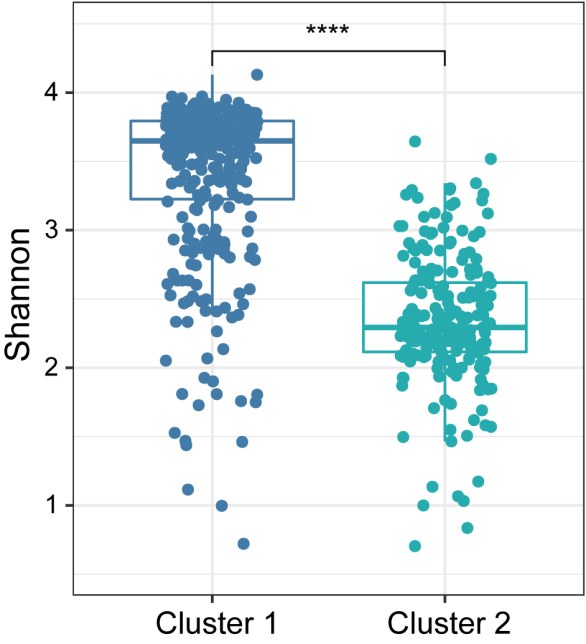


**Fig. S1. Comparison of the Shannon index between cluster 1 and cluster 2.** The Shannon index of cluster 1 was significantly higher than that of cluster 2. **** indicate that p < 0.0001


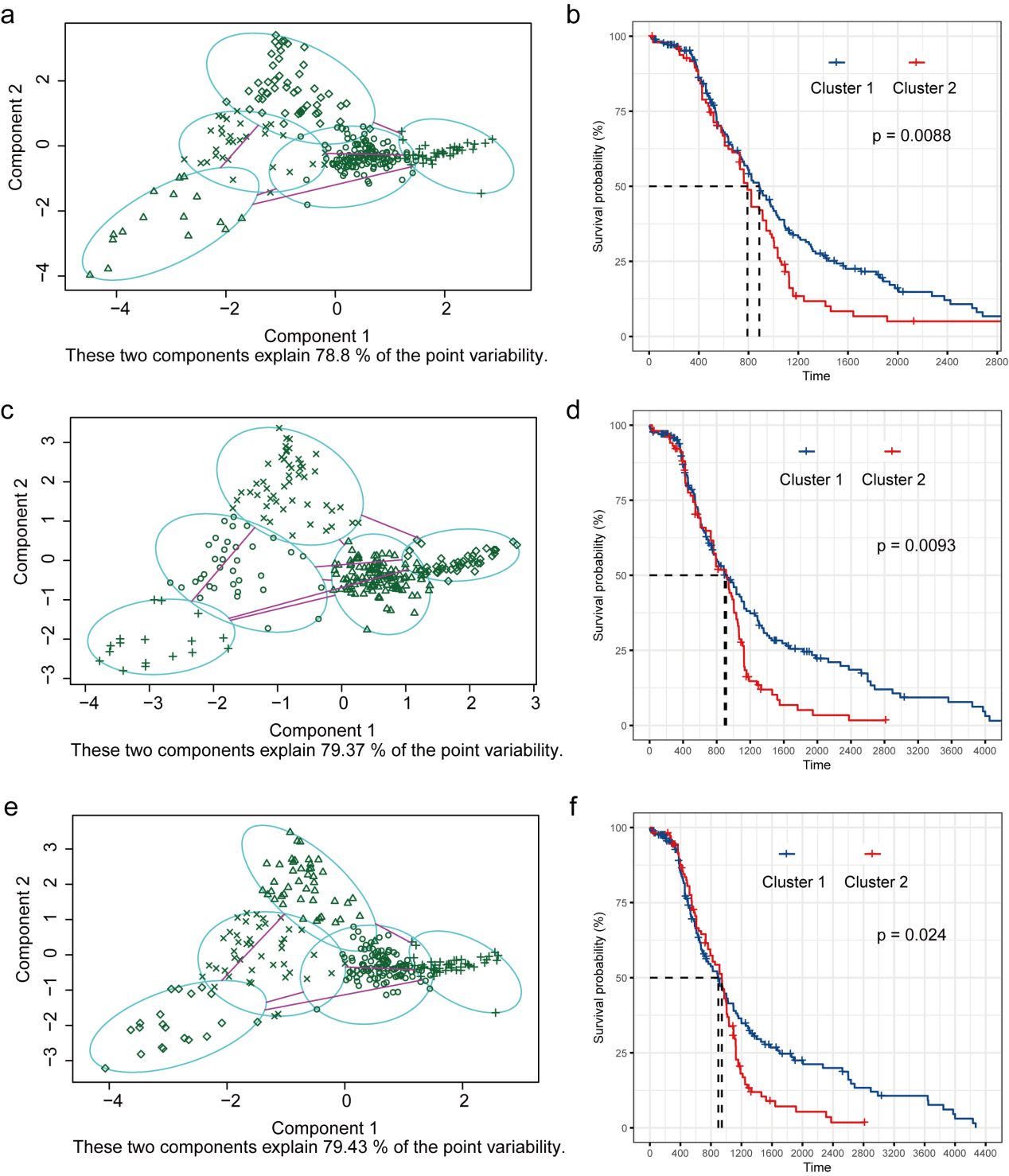


**Fig. S2. The stability of clustering was verified by multiple random sampling.** (a,c, and e) 50% of the samples were randomly selected for several times, and then PAM clustering based on tissue microbial abundance was performed. (b, d, and f) The five PAM clusters were divided into two groups, and the KM survival curve showed the difference in survival between the two groups.


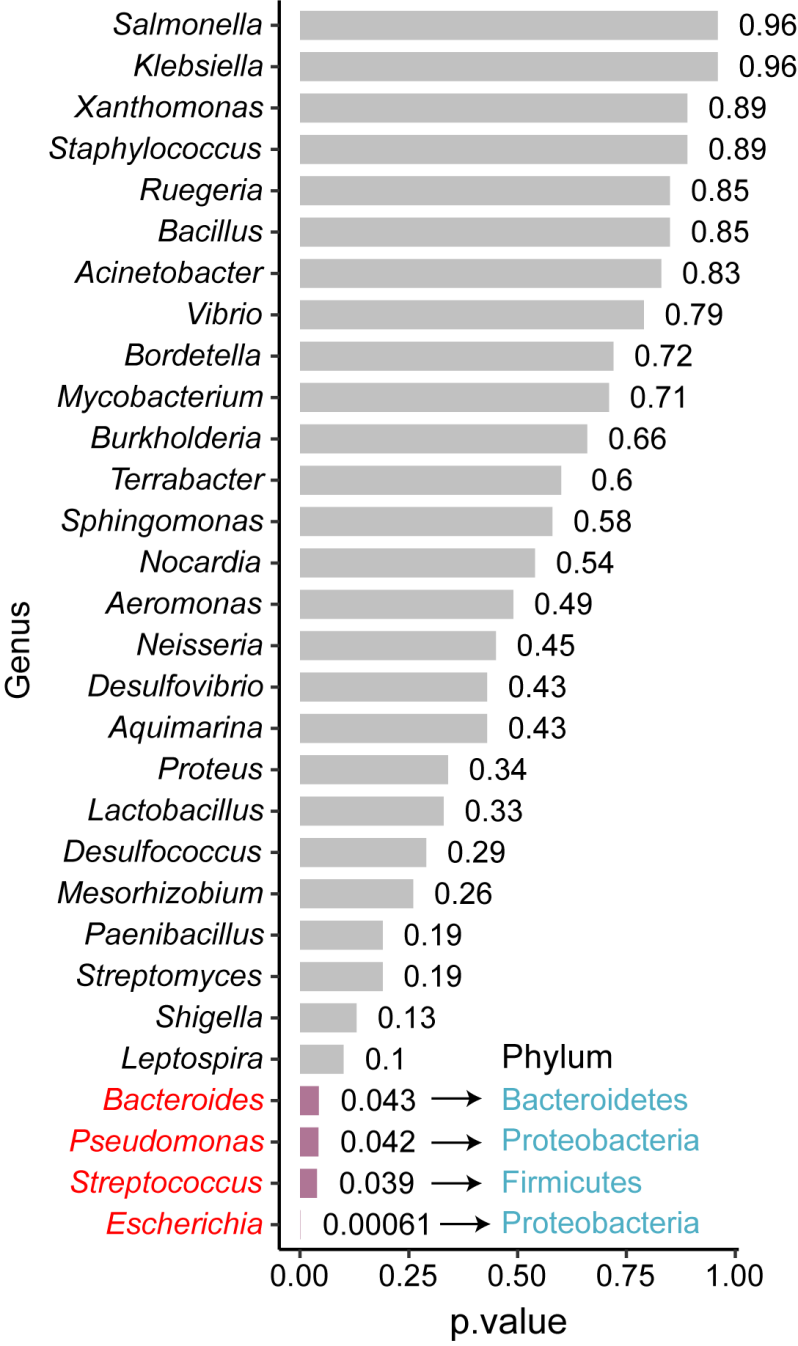


**Fig. S3. Univariate Cox regression analysis based on the genus level species.** Four genera were significantly correlated with CRC patient survival (p < 0.05). The species that was significantly correlated with patient survival was in red. Species in blue indicated the phyla that the genera belong to.
